# Lysinuric protein intolerance presenting as pancytopenia and splenomegaly mimicking acute leukaemia: a case report

**DOI:** 10.1186/s12887-023-04207-7

**Published:** 2023-08-01

**Authors:** Chanika Lokuhewage, Hashan Pathiraja, Piyumi Madawala, Sudarshana Bandara, Sachith Mettananda

**Affiliations:** 1 Colombo North Teaching Hospital, Ragama, Sri Lanka; 2 Department of Paediatrics, Faculty of Medicine, University of Kelaniya, Thalagolla Road, Ragama, 11010 Sri Lanka

**Keywords:** Lysinuric protein intolerance, Splenomegaly, Pancytopenia, Leukaemia

## Abstract

**Background:**

Lysinuric protein intolerance is a rare inherited metabolic disease due to autosomal recessive mutations of the *SLC7A7* gene. The affected patients commonly present with protein-rich food intolerance, failure to thrive, hepatosplenomegaly, muscle hypotonia and lung involvement due to impaired intestinal absorption and excessive urinary excretion of dibasic amino acids. Presentation with splenomegaly and cytopenia without other features has not been reported. Here we report a Sri Lankan girl with lysinuric protein intolerance presenting with pancytopenia and splenomegaly mimicking acute leukaemia.

**Case presentation:**

Two years and six months old Sri Lankan girl presented with persistent pancytopenia following a viral illness. She was asymptomatic without vomiting, diarrhoea, abdominal pain or irritability. Physical examination revealed pallor and isolated firm splenomegaly of 2 cm. Growth parameters and other system examinations were normal. Full blood count revealed anaemia, leukopenia and thrombocytopenia. The blood picture showed a mixture of hypochromic microcytic and normochromic normocytic red cells with occasional pencil cells and macrocytes. Bone marrow examination was normal except for occasional megaloblasts; however, serum vitamin B12 and red blood cell folate were normal. The metabolic screen showed a high anion gap compensated metabolic acidosis, high lactate and ketosis. Genetic mutation analysis using whole exome sequencing revealed compound heterozygous variants of the *SLC7A7* gene, confirming the diagnosis of lysinuric protein intolerance.

**Conclusion:**

We report a child with lysinuric protein intolerance presenting with pancytopenia and splenomegaly without other disease features. This case report adds to the heterogenic presentations of lysinuric protein intolerance, which is considered a multifaceted disease.

## Background

Lysinuric protein intolerance (LPI) is a rare inherited metabolic disease resulting from autosomal recessive mutations involving the *SLC7A7* gene [1]. It is characterised by impaired intestinal absorption and excessive urinary excretion of dibasic amino acids, especially lysine. The disease has variable presentations that include failure to thrive, protein-rich food intolerance, hepatosplenomegaly, muscle hypotonia, lung involvement, osteoporosis, seizures, and coma [2]. However, presentation with splenomegaly and cytopenia mimicking haematological malignancy without other features has not been reported. Here we report a Sri Lankan girl with LPI presenting with pancytopenia and splenomegaly mimicking acute leukaemia.

## Case presentation

Two years and six months old Sri Lankan girl presented with persistently low haemoglobin, white cell count and platelet count for two weeks following a viral illness. She was born to non-consanguineous healthy parents at term with a birth weight of 2.58 kg following an antenatal period complicated with pregnancy-induced hypertension of the mother. The child had a large ventricular septal defect at birth which was surgically closed at two months. She had two previous hospital admissions for respiratory tract infections, which had responded to standard antibiotics. Her development and immunisation have been up to date. Her parents were from a higher middle-class family, and she had been taking a well-balanced normal diet adequate in calories and proteins. She was asymptomatic without vomiting, diarrhoea, abdominal pain or irritability.

On examination, she had mild pallor but did not have jaundice, cyanosis, bleeding manifestations, generalised lymphadenopathy or oedema. Her weight was 11 kg (between − 1SD to -2SD), height was 85 cm (between − 1SD to -2SD), and the weight for height was between median and − 1SD. She did not show features of micronutrient deficiency. Abdominal examination revealed firm splenomegaly of 2 cm without hepatomegaly or other abnormalities. The examination of her cardiovascular, respiratory, and neurological systems was clinically normal.

Full blood count revealed haemoglobin of 9.3 g/dL, mean corpuscular volume of 72fL, white cell count of 1.7 × 10^9^/L (neutrophils-33% and lymphocytes-62%) and platelet count of 56 × 10^9^/L. The blood picture revealed a mixture of hypochromic microcytic and normochromic normocytic red cells with occasional pencil cells and macrocytes. The reticulocyte count was 1% and the direct agglutinin test was negative. Bone marrow aspirate and trephine biopsy revealed normocellular marrow, normoblastic erythropoiesis with occasional megaloblasts, granulopoiesis with normal maturation and < 1% blasts and normal megakaryopoiesis. There were no abnormal cells. Her serum ferritin was 5.7ng/mL (normal 15–200), serum B12 was 550pg/mL (normal 187–883) and red blood cell folate was 355ng/mL (normal 126–651). Abdominal ultrasonography revealed mild splenomegaly with a spleen length of 8.6 cm.

As the cause for splenomegaly was unexplained, further investigations to exclude a metabolic disease were carried out. Venous blood gas revealed a partially compensated metabolic acidosis with a pH of 7.40 and bicarbonate of 14mmol/L. Serum sodium was 142mmol/L, potassium was 4mmol/L, chloride was 105mmol/L, and the calculated anion gap was 27mmol/L (normal 8–16). Serum lactate was 3.1mmol/L (normal < 2), and urine was positive for ketones. Plasma ammonia, creatinine, glucose, cholesterol, and transaminases were normal. Viral studies for hepatitis B, hepatitis C, cytomegalovirus, Epstein-Barr virus and human immunodeficiency virus were negative and antinuclear antibody and lactate dehydrogenase levels were normal. Plasma amino acid profile was normal; however, urine organic acid profile revealed marked elevations of ketotic markers (acetoacetate and 3-hydroxybutyric acid) and moderate elevation of tricarboxylic acid cycle compounds (2-ketoglutarate, aconitic acid, and citric acid) during fasting as well as the fed state. Due to abnormalities in metabolic markers, genetic mutation analysis using whole exome sequencing was performed. This revealed compound heterozygous variants of the *SLC7A7* gene (NM_001126105.2:c.931 A > G and NM_001126105.2:c.1095 + 6T > C), confirming the diagnosis of LPI. Genetic mutation analysis of parents was not done due to the high cost.

She was started on citrulline 100 mg/kg/day with dietary protein restriction. She was also given oral iron (6 mg/kg/day of elemental iron). Oral folic acid (1 mg od) and intramuscular vitamin B12 treatment, which was commenced due to megaloblastic erythropoiesis in bone marrow, was discontinued following receipt of the genetic report. At the 3-month follow-up, she was clinically well with persistent splenomegaly of 3 cm. Her haemoglobin and white cell count had normalised; however, the thrombocytopenia was persistent, with a platelet count of 83 × 10^9^/L. At the 12-month follow-up, she continued to have splenomegaly of 3 cm and thrombocytopenia (platelet count 90 × 10^9^/L). Her haemoglobin and white cell count were 12.3 g/dL and 6.4 × 10^9^/L, respectively.

## Discussion and conclusions

LPI is an extremely rare autosomal recessive genetic disease characterised by the inability to absorb specific cationic dibasic amino acids that include lysine, arginine, and ornithine [3]. The genetic mutations are mapped to the *SLC7A7* gene, which encodes for the y + L-type amino acid transporter 1 (y + LAT1) subunit of a cationic amino acid transporter which is found primarily in the basolateral membrane of polarised cells such as intestinal and renal tubular cells [4, 5]. Additionally, y + LAT1 is reported to be present in non-polarised cells such as lymphocytes and macrophages [6].

Lysine is an essential amino acid important for proper growth and the production of carnitine, which is involved in fatty acid metabolism [7]. Arginine and ornithine are urea cycle intermediates [1]. The inability to absorb lysine, arginine and ornithine leads to classical features of LPI, which include protein-rich food refusal, vomiting, diarrhoea and failure to thrive. Other reported features include hepatosplenomegaly, renal failure, osteoporosis, sparse brittle hair, thin extremities with centripetal adiposity, and moderate intellectual disability [2, 8].

The patient described in this case report did not show any of the classic features of LPI but presented with splenomegaly and pancytopenia mimicking acute leukaemia. Although haematological abnormalities that include anaemia, leukopenia, and thrombocytopenia are reported in LPI, they have been linked to haemophagocytic lymphohistiocytosis (HLH) [5, 9]. HLH is a disease characterised by fever, splenomegaly, pan or bi-cytopenia, hypertriglyceridemia, hypofibrinogenemia, hyperferritinemia, and haemophagocytosis in the bone marrow [2]. A retrospective cohort analysis of sixteen patients with LPI showed that five patients fulfilled the criteria for the diagnosis of HLH. The reported prevalence of features of HLH was anaemia (7/16), thrombocytopenia (5/16), hepatosplenomegaly (16/16), hypertriglyceridemia (10/16), hypofibrinogenemia (3/16) and hyperferritinemia (11/12) [1]. In contrast, our patient did not show features of haemophagocytic lymphohistiocytosis, such as in haemophagocytic cells in the bone marrow and elevated serum ferritin, lactate dehydrogenase, triglycerides or hepatic transaminases.

Another unusual feature in this case report is the presence of high-anion gap metabolic acidosis. Although this can be attributed to the increased lactate and ketones found in this child, the exact relationship between the molecular defect and lactic acidosis and ketosis is unclear. Since arginine and ornithine are urea cycle intermediates, deficiency of these amino acids manifests as urea cycle defects. Although hyperammonaemia is the commonest metabolic abnormality, urea cycle defects can sometimes result in lactic acidosis [10]. Alternatively, a defect of the dibasic amino acid transporter at the basolateral membrane of the renal tubular epithelial cells and other non-polarised cells could lead to intracellular accumulation of lysine. Lysine, which is cytotoxic, could lead to lactic acidosis in cells [8].

Although hepatosplenomegaly is reported in patients with LPI, its mechanism is unclear. One possible mechanism is a defective y + LAT1 subunit which is also present in lymphocytes. Since the white pulp of the spleen consists of both T and B lymphocytes, defective expression of the y + LAT1 subunit of the amino acid transporter in the lymphocytes could be linked to splenomegaly in LPI [7].

In conclusion, we report a child presenting with pancytopenia and splenomegaly without other classical features of LPI mimicking acute leukaemia. This case report adds to the heterogenic presentations of LPI, which is considered a multifaceted disease [1]. Also, it highlights the importance of performing timely genetic diagnosis when children present with subtle clinical features that could not be matched accurately to a known clinical entity.

## Data Availability

Not applicable.

## Abbreviations

LPI: Lysinuric protein intolerance
LAT1: L-type amino acid transporter 1

## References

[1] Mauhin W, Habarou F, Gobin S, Servais A, Brassier A, Grisel C, Roda C, Pinto G, Moshous D, Ghalim F (2017). Update on lysinuric protein intolerance, a multi-faceted disease retrospective cohort analysis from birth to adulthood. Orphanet J Rare Dis.

[2] Kliegman RM, Geme JS (2020). Nelson textbook of pediatrics.

[3] Olgac A, Yenicesu I, Ozgul RK, Biberoğlu G, Tümer L (2020). Lysinuric protein intolerance: an overlooked diagnosis. Egypt J Med Hum Genet.

[4] Carpentieri D, Barnhart MF, Aleck K, Miloh T, deMello D (2015). Lysinuric protein intolerance in a family of Mexican ancestry with a novel SLC7A7 gene deletion. Case report and review of the literature. Mol Genet Metab Rep.

[5] Alqarajeh F, Omorodion J, Bosfield K, Shur N, Ferreira CR (2020). Lysinuric protein intolerance: pearls to detect this otherwise easily missed diagnosis. Transl Sci Rare Dis.

[6] Noguchi A, Takahashi T (2019). Overview of symptoms and treatment for lysinuric protein intolerance. J Hum Genet.

[7] Matthews DE (2020). Review of lysine metabolism with a focus on humans. J Nutr.

[8] Riccio E, Pisani A (2014). Fanconi syndrome with lysinuric protein intolerance. Clin Kidney J.

[9] Doireau V, Fenneteau O, Duval M, Perelman S, Vilmer E, Touati G, Schlegel N, Ogier de Baulny H (1996). Lysinuric dibasic protein intolerance: characteristic aspects of bone marrow involvement. Arch Pediatr.

[10] Guerrero RB, Salazar D, Tanpaiboon P (2018). Laboratory diagnostic approaches in metabolic disorders. Ann Transl Med.

